# Editorial for the Special Issue “Microsystem for Electronic Devices”

**DOI:** 10.3390/mi14040720

**Published:** 2023-03-24

**Authors:** Xinrui Ding

**Affiliations:** Key Laboratory of Surface Functional Structure Manufacturing of Guangdong High Education Institutes, South China University of Technology, Guangzhou 510641, China; dingxr@scut.edu.cn

The field of microsystems is a rapidly evolving area with a wide range of applications in the field of electronics [1,2,3]. From light-emitting devices and sensors to detectors and transistors, microsystems are playing an increasingly important role in our daily lives. However, the development of microsystems is not without its challenges. Interdisciplinary scientific problems, such as manufacturing, measurement, control, reliability, efficiency and sensitivity, must be solved in order to fully realize the potential of these tiny devices. Despite these challenges, the unique characteristics that arise due to the scale effect make microsystems an exciting area of research. With the advent of advanced manufacturing methods, the possibilities for novel microsystems and applications only continue to grow. In this Special Issue, we invited contributions that explored the latest developments in the field of microsystems and their applications in electronics.

This Special Issue includes 12 high-quality papers focused on the development of microsystems. Half of the published papers focus on light-emitting diode (LED) microsystems and related microelectronic systems [4,5,6,7,8,9], three papers discuss the cooling techniques of electronic microsystems [10,11,12], and the final three papers present several mechanical microsystems [13,14,15].

In LED microsystems and related microelectronic systems, Rashid et al. [4] conducted a simulation of a SiC-based LED device with a unique structural configuration of 4H-SiC and 6H-SiC layers, using a commercially available semiconductor device simulator. This yielded promising results in terms of luminous efficiency (25%) and external quantum efficiency (16.43%), with the potential for the customization and future development of efficient and cost-effective SiC-based LEDs through the direct bonding of SiC-SiC wafers. We [5] developed a phosphor converter based on a micro-angle tunable tilted filter for hybrid-type laser lighting devices to address the issue of carbonizing under high-energy density in silicone phosphor converters. The filter and scattering characteristics of phosphors generate two luminous areas on the converter, and the lighting effects can be adjusted using tilt angles. When the tilt angle is 20°, the luminous flux increases by 11.5% and the maximum temperature reduces by 22.8% under the same luminous flux and correlated color temperature of 6500 K. Yu et al. [6] proposed micro-prism patterned remote phosphor films, made by large-scale roll-to-roll imprinting, to significantly improve the light and color performance of LEDs. The micro-prism film on the incident surface of the remote phosphor film extracts backward light through a double reflection and the micro-prism film on the exit surface retains blue light inside the remote phosphor film to enhance phosphor excitation. This technique represents a promising direction for fabricating high-performance microstructure-based color converters. Guo et al. [7] fabricated flip-chip light-emitting diodes with a composite microstructure comprising Ag/SiO_2_/distributed Bragg reflector/SiO_2_ to enhance light extraction efficiency and vertical light output for high-power applications. The composite reflector exhibits a higher reflectance compared to the commonly used Ag-mirror reflective structure, which is due to the increased reflective area in the sidewall and partial area of the n-type gallium nitride contact orifices. As a result, the light output power of these diodes was found to be 6.3% higher, and the external quantum efficiency was improved by 6.0% at 1500 mA compared to traditional light-emitting diodes. Zheng et al. [8] could regulate the correlated color temperature of white LEDs by modulating the transparence and haze of a paraffin–polydimethylsiloxane film. The results show that the correlated color temperature can be modulated from 15,177 K to 3615 K with a range of 11,562 K. Liu et al. [9] proposed a hybrid process of plasma oxidation and femtosecond laser ablation for the precision removal of copper in integrated circuits. The process oxidizes the surface copper layer to copper oxide and then removes it using a low fluence femtosecond laser without damaging the underlying copper, achieving a surface roughness of 3 nm and a removal accuracy of 4 nm.

On the cooling techniques of electronic microsystems, Chen et al. [10] developed multilayer copper micro-meshes as pool boiling enhancers for commercial compact electronic cooling with a maximum critical heat flux of 207.5 W/cm^2^ and a heat transfer coefficient of 16.5 W/(cm^2^·K) due to abundant micropores and capillary wicking, making them highly competitive for use in high-power cooling in commercial applications. The design and construction of microstructures in heat sinks has been shown to improve heat dissipation efficiency. Yuan et al. [11] conducted a study of four types of surface treatments on heat sinks, combined with thermal radiation coating, to examine the impact on thermal emissivity, surface roughness and heat dissipation. The results show that surface roughness could increase thermal emissivity by up to 2.5 times and the thermal radiation coating improved heat dissipation by enhancing heat conduction at the coating-heat sink interface. Fu et al. [12] proposed a novel vortex generator and studied its micro thermal-hydraulic performance in a heated tube, through both experimental and numerical investigations, with the results showing improved heat transfer and decreased friction, and with the maximum thermal enhancement factor of 1.21.

In mechanical microsystems, Wu et al. [13] developed micro-textured tools in dry milling of Ti-6Al-4V alloys by making textures on rake faces and filling with molybdenum disulfide. The results show that the new tools can reduce cutting forces, temperatures, and power consumption by approximately 15%, 10%, and 5%, respectively, and improve tool lives by 20–25% via self-lubricating function. A novel electrostatic self-excited resonator driven by DC voltage that can achieve variable velocity–position characteristics through pre-tension/pre-compression constraints was developed by Qi el al. [14]. The resonator can switch between pre-compression and pre-tension by adjusting the distance between two constraint bases, and thus controlling the position of the maximum velocity output of the oscillating beam. Zhong et al. [15] investigated the effects of solution and aging temperatures on the microstructure and mechanical properties of ultra-high strength stainless steel 10Cr13Co13Mo5Ni3W1VE (S280). The results show that S280 exhibits the best mechanical properties at a 1080 °C solution temperature and a 540 °C aging temperature. These optimal conditions result in fine and dispersed strengthening phases and the recovery of austenite in high-density dislocation martensite matrix, leading to good strength and toughness.

I would like to extend my gratitude to all the authors for their submissions to this Special Issue. Additionally, my appreciation goes to the reviewers for their dedication and efforts to improve the quality of the submitted papers.
